# Allelotyping identification of genomic alterations in rectal chromosomally unstable tumors without preoperative treatment

**DOI:** 10.1186/1471-2407-10-561

**Published:** 2010-10-18

**Authors:** Benoît Romain, Agnès Neuville, Nicolas Meyer, Cécile Brigand, Serge Rohr, Anne Schneider, Marie-Pierre Gaub, Dominique Guenot

**Affiliations:** 1 Service de Chirurgie Générale et Digestive, Hôpitaux Universitaires de Strasbourg, Hôpital de Hautepierre, Avenue Molière, 67098 Strasbourg Cedex, France; 2 Université de Strasbourg (UdS), EA 4438 Physiopathologie et Médecine Translationnelle, 3 Avenue Molière, 67200 Strasbourg, France; 3 Laboratoire d'Anatomie Pathologique, Hôpitaux Universitaires de Strasbourg, Hôpital de Hautepierre, Avenue Molière, 67098 Strasbourg Cedex, France; 4 Département de Santé Publique, CHRU, 67091 Strasbourg Cedex, France; 5 Laboratoire de Biochimie et Biologie Moléculaire, Hôpitaux Universitaires de Strasbourg, Hôpital de Hautepierre, Avenue Molière, 67098 Strasbourg Cedex, France

## Abstract

**Background:**

Numerous studies reported genomic alterations in colorectal human tumors but few focused on rectal tumors with the specification of preoperative-treated or untreated tumors. The goals of this study were to list chromosome allelic imbalances and correlate their frequency with tumor progression and to identify potential molecular markers of progression in rectal chromosomally unstable tumors without preoperative treatment.

**Methods:**

Genomic alterations of 57 rectal tumors assessed by allelotyping targeting 33 chromosomal loci, were clusterised and compared to those of 151 left colon tumors.

**Results:**

Clustering separated the rectal tumors without preoperative treatment into three subtypes according to the allelic imbalance frequency and genomic alteration associations. The tumors without preoperative treatment displayed a significantly higher allelic imbalance frequency (54%) than the tumors with preoperative treatment (33%), suggesting that treatment could target highly altered tumor clones. Interestingly, the survival analysis identified three potential prognostic molecular survival markers, D1S197, D5S430, and D14S65, for tumors without preoperative treatment.

**Conclusion:**

Based on the genomic status of 33 chromosomal loci, we observed that rectal tumors without preoperative treatment segregate according to the global allelic imbalance frequency but without correlation to the tumor progression. Moreover, the detailed associations of alterations in rectal tumors are different from those described in colon tumors suggesting that rectal and left tumors should be considered as separate entities. Finally, potential prognostic genomic molecular markers for survival are proposed which status could specify the clinical course of the tumors.

## Background

Colorectal cancer is the second most common cause of cancer deaths in the Western world after lung cancer for men and breast cancer for women [1]. In 2000, in France, the incidence of colorectal cancer was 36 300 patients, and there are approximately 12 000 new cases of rectal cancers diagnosed per year [2,3].

A multistep process involving mutational events in both oncogenes and tumor suppressor genes is now accepted worldwide for the development of most cancers. In the current model of the stepwise progression of colorectal carcinogenesis [4], each step is initiated by the acquisition of additional genomic abnormalities that confer a growth advantage to the targeted cell. Because of the increasing complexity of the combination of alterations observed during tumor progression, understanding the mechanisms underlying the recurrences and invasiveness of early-stage cancers remains of critical importance [5]. Although these chromosome alterations have been extensively analyzed with genome-wide chromosomal approaches, such as comparative genomic hybridization (CGH), reliable genomic markers for characterizing the progression to malignant stages or the resistance to treatment have yet to be proposed for colorectal cancer. Currently, the only marker with sufficient evidence to justify routine clinical assessment is *KRAS *mutational analysis for EGFR-specific therapy decision-making [6]. For a better validation, improvement in the tumor inclusion could be made, for example, by discriminating the carcinoma localization or phenotype. Indeed, several studies have suggested that carcinomas from proximal, distal, or rectal sites [7] as well as cancers of the microsatellite (MIN) and chromosomal unstable (CIN) phenotype [8] have different properties and should consequently be analyzed separately.

Surgery with total mesorectal excision with preservation of pelvic nerves is the mainstay of treatment, and neoadjuvant chemoradiotherapy is presently considered as the standard treatment of most T3-4 rectal cancers. In France, a combination of radiotherapy (45 Gy/5 weeks) with concurrent capecitabine (1,600 mg/m^2^) is the most popular protocol [9]. Preoperative treatment significantly decreases the local recurrence rate, but has no effect on the global survival and the metastasis progression rate [10].

In clinico-pathological and molecular studies, the rectum is often considered as a part of the distal colon rather than a separate entity. The few studies that analyzed mutations in key regulatory oncogenes and tumor suppressor genes for sporadic cancer sites showed a significant difference in mutation rates for K-Ras, TP53, and APC, specifically between the rectum and the rest of the colon [11,12]. However, the main difficulty inherent to the molecular study of rectal tumors is that they are often irradiated before surgery, leading to potential genomic abnormality selection, and, to our knowledge, the effects of radiochemotherapy on the molecular phenotype of the tumors is not well described [13].

The aim of our study was to determine whether the frequency of genomic alterations could be correlated to the tumor progression and to identify potential molecular markers for survival in a homogeneous cohort of rectal chromosomally unstable tumors without preoperative treatment. In addition we aimed to compare the genomic profiles of left and rectal tumors.

The tumor genomic alterations were evidenced by means of the allelic imbalance at 33 microsatellites (MS) mainly localized in chromosomal regions previously described as being frequently altered in colorectal cancer [14,15]. Allelotyping, based on a sensitive and automated fluorescent-based DNA technology [16], is a fast, cheap, and sensitive approach used in routine clinical diagnosis.

Using allelotyping and clustering of the data allowed us to show that rectal tumors without preoperative treatment segregate into three sub-types according to the global allelic imbalance frequency but without correlation to the tumor progression. Also, our data revealed that according to the global AI frequency, left colon tumors were distributed similarly to rectal tumors without preoperative treatment, but were separated according to the nature of the loci associated in the cluster trees. These results reinforce the concept that colon and rectal tumors are distinct entities.

## Methods

### Patients and tumor specimens

Tumor specimens and the clinical data obtained from 119 consecutive rectal tumors (83 men and 36 women with an average age of 66.5 years (from 24 years to 89 years)) were obtained from the Surgical Department at the University Hospital Hautepierre (Strasbourg-France) from December 1996 to August 2006 according to the French Ethical Committee recommendations. Staging, angioinvasion, stromal inflammation and differentiation of the tumors were evaluated at the Pathology Department, after resection for tumors without preoperative treatment and after the neoadjuvant therapy and resection for tumors with preoperative treatment. For tumors without preoperative treatment, each sample contained at least 40% of tumor cells as estimated by allelic imbalance at loci known to be deleted (TP53, APC, 18q) and by histopathological examination. All rectal tumors were characterized through the use of endorectal echo-endoscopy and/or a pelvic MRI, a thoraco-abdominal scanner, or abdominal echography with a Thorax X-Ray and peroperative observations. An emergency surgery was carried out for 3 patients out of 119 (2.5%) for an occlusive syndrome; in the other cases, the diagnosis was confirmed with biopsies during colonoscopy. The median follow-up was 25 months.

Allelotyping was performed for 57 patients (40 men and 17 women) out of the 119, with an average age of 66 years (from 34 years to 87 years of age). Of the 57 patients, 33 had no preoperative treatment and 24 had undergone preoperative treatment (preoperative radiotherapy, n = 16; preoperative radiochemotherapy, n = 8; Table 1). No biopsies of the tumors before treatment were available. To compare the distribution of genomic alterations between left colon and rectal tumors, a cohort of 151 left CIN colon tumors were included whose TNM stages are indicated in Table 2.

**Table 1:** Clinical and anatomo-pathological characteristics of rectal tumors analyzed for 33 microsatellites

				**With preoperative treatment (33 MS allelotyping) n = 24**	
		
		**Total tumors n = 119**	**Without preoperative treatment (33 MS allelotyping) n = 33**	**With preoperative radiotherapy n = 16**	**With preoperative radio-chemotherapy n = 8**
		
Age	Median	66.5 years	69 years	68 years	61 years
	Range	[24; 89]	[34; 87]	[44; 82]	[42; 68]
Sex Ratio	Male	83	22 (66.7%)	11 (68%)	7 (87.5%)
	Female	36	11 (33.3%)	5 (32%)	1 (12.5%)
UICC Staging	0-I	34	12	3	
	II	33	6	6	
	III	25	8	4	
	IV	27	7	11	
Localization	[0; 5 cm]	53	9	11	3
	]5; 10 cm]	44	13	5	5
	]10; 15 cm]	22	11	0	0
Stromal Inflammation*	No inflammation	81 (68.1%)	26 (78.8%)	14 (58.3%)	
	Inflammation	38 (31.9%)	7 (21.2%)	10 (41.6%)	
Differenciation*	Not differentiated	4 (3.3%)	0 (0%)	2 (8.3%)	
	Middle differentiated	39 (32.8%)	13 (39.4%)	7 (29.3%)	
	Well differentiated	76 (63.9%)	20 (60.6%)	15 (62.5%)	
Angio-invasion*	No angio-invasion	101 (84.9%)	26 (78.8%)	6 (25%)	
	Angio-invasion	18 (15.1%)	7 (21.2%)	18 (75%)	
Local recurrence rate at 2 years		8.3%	6.1%	8.3%	
Metastatic relapse at 2 years		22.7%	18.1%	29.2%	
2 years overall survival		91%	89%	81%	

MS = microsatellite, UICC = Union International Against Cancer - * These items were evaluated after resection for tumors without preoperative treatment and after neoadjuvant therapy and resection for tumors with preoperative treatment.

**Table 2:** Characteristics of the 151 patients with left colon tumors

		**Left colon tumors**
		
Age	Median	66 years
	Range	[37; 88]
Sex Ratio	Male	90 (59.6%)
	Female	61 (40.4%)
Stage UICC	0-I	22 (14.6%)
	II	31 (20.5%)
	III	27 (17.9%)
	IV	71 (47%)

The total dose of preoperative radiotherapy (RT) was 39 Gy in 13 fractions over 3 weeks. The dose per fraction was 3 Gy. For patients who underwent radiotherapy and concurrent chemotherapy (CT), the first CT cycle was administered from days 1 to 5 of the RT treatment. Fluorouracil (FU) at a dose of 350 mg/m^2^/d was delivered for 20 minutes in 100 mL of saline infusion 1 hour before RT. All relevant clinical and pathological characteristics were collected and entered into a retrospective database.

### DNA extraction and microsatellite amplification

DNA was extracted and amplified from patients' blood, frozen tumors, and normal mucosa sections by fluorescence PCR as described previously [17]. The panel of 33 MS targeting 17 chromosomes [18] allows for the detection of at least 1 alteration in each tumor tested and corresponds to the most frequently rearranged loci in colon cancer [14,15]. Primers were obtained from either the Genome DataBase http://www.gdb.org or Genmap'99 http://www.ncbi.nlm.nih.gov/genemap99/. Amplified fragments were analyzed on an ABI Sequencer (Applied Biosystems(r), France SA) allowing a sensitive and quantitative evaluation of the allele ratio by measuring the peak height of both alleles. AIs were confirmed by a second independent PCR. Cut-off values for significant AI were determined from previous studies [17,19]. The allelotyping detects a change in the allele ratio in the carcinoma relative to the allele ratio in paired normal blood cells and normal mucosa [18]. A modification of the allele ratio in the carcinoma relative to the matched control is referred to as AI, and the presence of an additional peak is referred to as MSI. The locus AI frequency corresponds to the percentage of tumors bearing an alteration at a given locus within a cohort, whereas the mean global AI frequency corresponds to the number of informative loci that are altered within a cohort.

In order to exclude carcinomas with an MSI-H phenotype, the panel of five MS recommended by the U.S. National Cancer Institute (BAT25, BAT26, D5S346, D2S123, and D17S250) was included in the analysis [20]. Homozygous, non-amplified, and MSI microsatellites were considered as non-informative.

### Statistical analyses

The variables were studied using χ^2 ^and Fisher's exact tests for qualitative variables and the Student's *t*-test for quantitative variables. Survival analyses were analyzed by a Kaplan-Meier test and no correction for multiple testing was applied. The total threshold of significance was fixed at 5%. To assess the independence of the prognosis value of the survival markers, the Cox proportional hazards model was fitted with a stepwise backward method and with adjustment for confounding variables. For clustering analysis, data were coded in binary form using "1" for a locus in AI and "0" for a normal informative locus. Homozygous were treated as missing data. Data were then clustered using a two-way unsupervised clustering method and the analyses were run using the uncentered correlation metric with complete linkage clustering. No bootstrap method was used to assess the stability of clusters. This analysis was carried out using the Cluster 3.0 software. The cluster trees were produced using Java Tree View 1.0.4 (Eisen's Softwares(r)). All statistical analyses were performed with the Statistical Package for Social Sciences software (SPSS for Windows version 11.5).

## Results

### Mean global AI frequency

Clinical data for 119 consecutive rectal tumors were retrospectively analyzed. TNM stages, distribution, histological data (stromal inflammation, differentiation, angio-invasion), local recurrence rate, and two-year overall and progression-free survivals (PFSs) were in accordance with the literature [21]. The global local recurrence rate at two years was 8.3% (6.9% for patients without preoperative treatment and 9.7% for patients with preoperative treatment); however, this difference was not significant. The 2-year global survival rate was 91% for all patients. The 2-year progression-free survival rate was 81% for patients with tumor stages inferior or equal to III. Out of 119 tumors, 2 were MSI-H (1.7%) and were excluded from the analysis.

**Figure 1:**
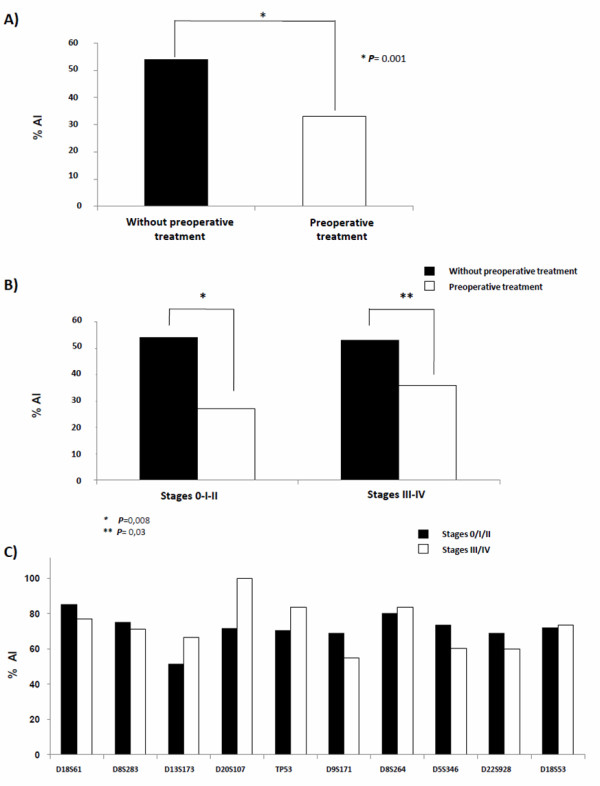
**Allelic imbalance frequency distribution**. A) Global AI frequency in tumors without (n = 33) or with preoperative treatment (n = 24). B) AI frequency for tumors without or with preoperative treatment according to TNM stages (UICC). C) AI frequency function of tumor TNM stages according to the 10 most frequently altered loci in rectal tumors without preoperative treatment (n = 33).

Allelotyping was performed for 33 microsatellites on 57 rectal tumors of the CIN phenotype and all clinical stages. Twenty-four tumors had preoperative treatment and 33 tumors had no preoperative treatment. The mean global AI frequency per tumor was 45% in the cohort of 57 rectal tumors, and a significant decrease in the frequency was observed between rectal tumors without preoperative treatment (mean AI frequency 54%) and rectal tumors with preoperative treatment (mean AI frequency 34%, *P *= 0.001, Figure 1A). This difference remained significant (39%, *P *< 0.05) after removing tumors that had lost all AIs after preoperative treatment (n = 4/24). The number of tumors with at least one MSI microsatellite was 26 out of 33 tumors without preoperative treatment (mean 5%) and 7 out of the 24 tumors with preoperative treatment (mean 4%).

#### Mean global AI frequency and clinical stages

**Figure 2:**
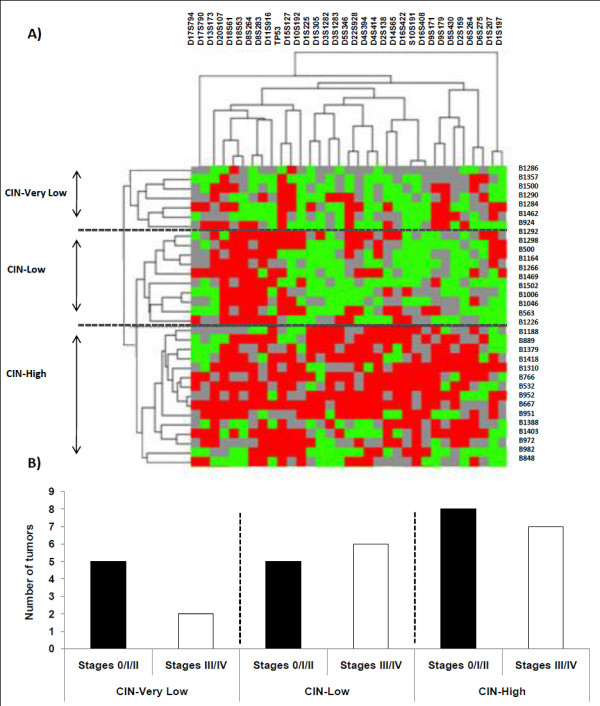
**Two-way hierarchical clustering of allelotyping 33 rectal tumors**. A) Clustering of 33 rectal tumors without preoperative treatment. Vertical columns correspond to microsatellites, horizontal lines correspond to tumors. Red squares correspond to loci with allelic imbalance, green squares to normal loci, and grey squares to non-informative. B) Stage distribution according to clusters CIN-VL, -L, and -H (n = 33).

No significant difference in the mean AI frequency was observed between early (0/I/II) and late (III/IV) TNM stages within each group of tumors (55% for stages 0/I/II vs. 52% for stages III/IV without preoperative treatment and 24% for stages 0/I/II vs. 33% for stages III/IV with preoperative treatment; Figure 2B). However, when comparing tumors with or without preoperative treatment, the mean global AI frequency was significantly different for stages 0/I/II (*P *< 0.05) and stages III/IV (*P *< 0.05; Figure 1B).

### Loci involved in tumor progression

In order to identify loci involved in the progression of tumors without preoperative treatment, the locus AI frequency was analyzed for each microsatellite according to the stage of the tumors. The mean locus AI frequency ranged from 25% to 82%. The 10 most altered loci displayed AI frequencies in excess of 60%: D5S346 (5q), D8S264 (8p), D8S283 (8p), D9S171 (9p), D13S173 (13q), TP53 (17p), D18S53 (18p), D18S61 (18q), D20S107 (20q), and D22S928 (22q) (Figure 1C), but no significant correlation with the tumor stage was found.

### Clustering analysis of genomic alterations for tumors without preoperative treatment

To further characterize the associations of alterations involved in tumor progression, we performed a two-way hierarchical unsupervised clustering analysis of genomic alterations in rectal tumors without preoperative treatment. This analysis indicated three clusters of tumors with distinct levels of CIN (Figure 2A). One cluster (CIN Low or CIN-L) had tumors harboring a low number of alterations: on average 43% of the loci were altered (27%-71%; n = 11), and they were mainly altered at markers on 8p (D8S264), 17p (TP53, close to the p53 gene), 18q (D18S61, D18S53), and 20q (D20S107). A second cluster of tumors (CIN-High or CIN-H) harbored a large number of alterations: on average 72.8% of the loci were altered (38%-100%; n = 15), and the alterations were widely distributed among all MS, but also included alterations on 8p, 17p, 18q, and 20q. A third cluster (CIN-very Low or CIN-VL) included tumors with a very low number of alterations: on average 29% of loci were altered (14%-48%; n = 7). The mean number of loci altered was significantly different between the three clusters (CIN-H, -L, -VL, *P *< 0.05), but within a cluster there was no significant correlation with a specific clinical stage (Figure 2B; 8 stages 0/I/II versus 7 stages III/IV in CIN-H; 5 stages 0/I/II versus 6 stages III/IV in CIN-L, and 5 stages 0/I/II versus 2 stages III/IV in CIN-VL) or with the global or progression-free survival.

It should be noted that the alterations of D5S346 (informative for APC) and TP53 (informative for p53) were not associated in the clustering tree.

### Survival studies and genomic alterations

Identification of loci whose status could potentially modulate global survival and progression-free survival was performed by Kaplan-Meier analysis of tumors with or without preoperative treatment.

#### Potential survival markers for tumors without preoperative treatment

**Figure 3:**
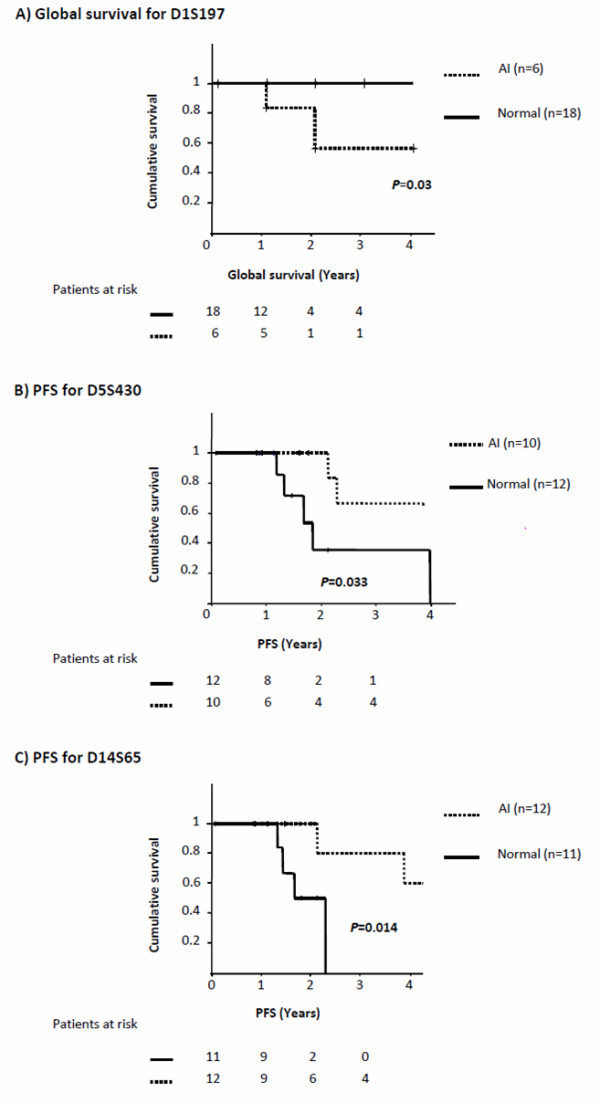
**Event-free survival computed using the Kaplan and Meier method**. The Kaplan-Meier survival curves were computed according to allelic imbalances at loci D1S197, D5S430, and D14S65 for patients without preoperative treatment. A) Global survival according to locus D1S197 status. B) PFS according to locus D5S430 status. C) PFS according to locus D14S65 status.

Among the 33 microsatellites analyzed for the 33 tumors without preoperative treatment, the status of D1S197, D5S430, and D14S65 significantly influenced survival. Tumors without AI at locus D1S197 had a overall survival that was significantly higher (*P *< 0.05) than those with AI (Figure 3A). Tumors with AIs at loci D5S430 and D14S65 had a PFS that was significantly higher (*P *< 0.05) than those with no AI (Figure 3B-C) (Table 3). Neither sexe nor age was a independent prognostic factor when adjusting the survival analysis of these microsatellites in overall and in PFS (*P *> 0.3).

**Table 3:** Distribution of prognostic molecular survival markers between patients with and without preoperative treatment

	**Without preoperative treatment** **(n = 33)**	**With preoperative treatment** **(n = 24)**
	
Global Survival	**D1S197**^a^	**D9S179**^b^
		**D17S790**^b^
		**D2S159**^a^
		
Progression Free Survival	D14S65^a^	**D2S159**^a^
	D5S430^a^	D6S264^a^

Loci with a good prognosis are indicated in bold when normal and are underlined when bearing AI.

^a ^p < 0.05;

^b ^p < 0.01

#### Potential survival markers for tumors with preoperative treatment

Among the 33 microsatellites analyzed for the 24 tumors with preoperative treatment, a significant difference in global survival associated with D2S159, D9S179, and D17S790 was observed (data not shown). Tumors with no AI at these loci displayed a better global survival than those with AIs at these loci (*P *< 0.05). On the other hand, tumors without an AI at locus D2S159 but with an AI at locus D6S264 had a better PFS (*P *< 0.05) (Table 3).

### Clustering analysis of 151 left allelotyped colon tumors

**Figure 4:**
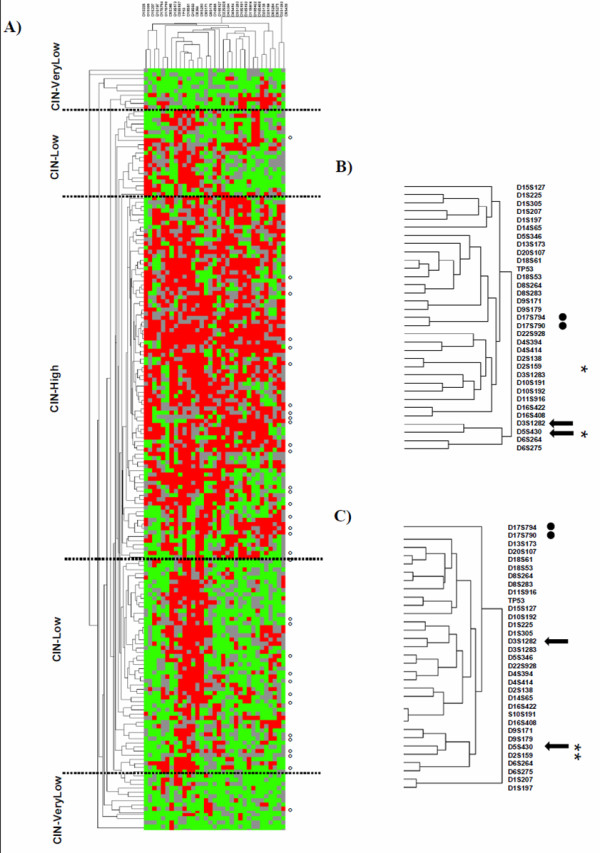
**Two-way hierarchical clustering of allelotyping rectal and colon tumors**. Vertical columns correspond to microsatellites, horizontal lines correspond to tumors. Red squares correspond to loci with allelic imbalance, green squares to normal loci, and grey squares to non-informative. A) Left colon tumors (n = 151) mixed with rectal tumors (n = 33). Dots correspond to rectal tumors without preoperative treatment. B) Dendrogram of left colon tumors (cluster not shown). C) Dendrogram of rectal tumors without preoperative treatment (from Figure 2). For B) and C) Arrows and dots: examples of loci associated in the left colon tumors but not associated in rectal tumors. Asterisks: loci associated in rectal tumors but not in left colon tumors.

To discriminate genomic alterations between left colon and rectal tumors without preoperative treatment, their mean global AI frequency and alteration distribution were compared. The mean global AI frequency was identical for the 151 left colon tumors and for rectal tumors (54% and 53.5%, respectively). The two-way hierarchical clustering separated left colon tumors into three clusters (CIN-H,-L, -VL; data not shown), as it did for rectal tumors. Within each cluster, the mean global AI frequency was similar for left colon and rectal tumors, but was significantly different between the clusters (*P *< 0.01). When left colon and rectal tumors were combined within the same cluster, the 2-way hierarchical clustering could not separate them (Figure 4A) and the mean locus AI frequency for each of the 33 microsatellites was not statistically different between both groups of tumors (χ^2 ^test).

As evidenced by the cluster trees and apart from the panel of markers located on 8p, 18q, 17p, and 20q, which aggregated as for the rectal tumors, combinations of loci were different between left colon and rectal tumors without preoperative treatment (Figure 4B-C). For example, loci D5S430 and D3S1282 or D17S790 and D17S794 were found to be associated in the clustering tree of left colon tumors, but were not associated in the rectal tumor tree; similarly, loci D5S430 and D2S159 were associated in the clustering tree of rectal tumors, but not in that of left colon tumors (Figure 4B-C).

## Discussion

The aim of the present work was to correlate the allelic imbalance frequency and tumor progression, and to compare AI distribution between left and rectal tumors. Despite the small size of the cohort, no other studies, to our knowledge, have analyzed a homogeneous collection of rectal tumors. We also tempted to identify potential molecular markers for survival in a cohort of rectal chromosomally unstable tumors without preoperative treatment.

Clustering of rectal tumors without preoperative treatment distinguished three subtypes of tumors according to the mean AI frequency at 33 chromosomal loci, but independent of the tumor stage. These results suggest that the potential for metastasis could be present very early in tumor development.

### Preoperative treatment and AI frequency

There was a significant decrease in the mean global AI frequency of tumors with preoperative treatment as compared with tumors without preoperative treatment, even when those with no more alterations after preoperative treatment were excluded. To explain these AI frequency differences between tumors with or without preoperative treatment, one could hypothesize that the clones that were sensitive to radiochemotherapy had a high level of alterations, i.e., a high number of altered chromosome loci. This suggests that within a tumor, the treatment will target some specific sensitive chromosomal loci. Unfortunately, biopsies of the tumors prior to the treatment were not available, and comparison of genomic alterations before and after treatment was not possible. Our observation is in agreement with Tannapfel et al. [13], who previously showed that the apoptotic index increased and the proliferative activity decreased significantly after radiochemotherapy in resected rectal tumors as compared to pretreatment biopsies. A focus on loci and their associated genes whose AI frequencies are significantly different before and after preoperative treatment would permit the specification of a set of alterations that predict the sensitivity of a tumor to treatment. Indeed, Tannapfel et al. [13] showed that p53-deleted tumors generally exhibited a higher apoptotic index. However, as in our cohort, the preoperative treatment did not efficiently eliminate clones with an altered TP53 locus (82% vs. 50% of tumors with an altered TP53 locus in tumors without and with preoperative treatment, respectively); the status of the TP53 locus did not specify the tumor sensitivity to preoperative treatment. However, preoperative treatment might decrease the global AI frequency by killing tumor clones bearing numerous alterations, while clones with a low level of alterations would resist the treatment.

On the other hand, although a correlation between the progression of the tumor stage and the increase in allelic imbalance was previously proposed [19,22], clustering of the allelotyping performed for the primary tumors and their matched metastasis showed that any tumor stage could be associated with any frequency [23]. Therefore, the number of genomic alterations could not predict the progression, and the invasive and metastatic potential are probably present at an early stage of tumor development.

### Altered loci and associated genes

We identified 10 loci that were altered in more than 60% of tumors with no preoperative treatment regardless of the tumor stage: D5S346, 5q; D8S264, 8p; D8S283, 8p; D9S171, 9p; D13S173,13q; TP53, 17p; D18S53, 18p; D18S61, 18q; D20S107, 20q; and D22S928, 22q.

Allelotyping identifies chromosome loci rather than specific genes and does not specify whether the alteration corresponds to a deletion or a gain of a locus. Despite these inconveniences, some of these loci target genes that have been shown to be implicated in colorectal carcinogenesis. It is well known that 18q is involved in tumor progression. DCC [24] and SMAD4 [25] are localized on 18q and are involved in colorectal carcinogenesis. Accordingly, we found that 18q was frequently altered in our cohort. Locus D18S61 (18q22) is close to the Bcl-2 gene that codes for an anti-apoptotic and oncogenic protein [26]. Conflicting data have been reported for Bcl-2, as Charara et al. [27] demonstrated that tumors overexpressing Bcl-2 displayed no residual tumor in response to neoadjuvant chemoradiotherapy, whereas Reerink et al. [28] found no relationship between tumor response in chemoradiotherapy and the level of Bcl-2 expression. This latter observation was corroborated in a recent meta-analysis of rectal tumors [29]. Evidence has been provided that another gene, GNAL (G protein alpha), localized on 18p close to D18S53, regulates several transforming functions that are linked to the acquisition of aggressive phenotypes in solid tumors [30].

The *TP53 *locus is informative for the p53 protein, which is called the cellular "gatekeeper" for growth and division [31], and this locus is lost early in the progression from adenoma to carcinoma [32]. *TP53 *has been investigated as both a prognostic factor and a predictor of the response to therapy, but with conflicting results, as the study design made it difficult to draw firm conclusions [27,33-35]. Therefore, the European Group on Tumor Markers ruled it as insufficient evidence and did not recommend a routine use for p53 as either a prognostic or predictive factor [36]. Indeed, in our cohort, although the altered TP53 frequency decreased after preoperative treatment, the AI frequency remained high (50%); thus, its status could not be predictive of the sensitivity to preoperative treatment. On the other hand, D5S346 is informative for the APC gene, which promotes the degradation of β-catenin and therefore limits the transcription of Wnt target genes involved in regulating the cell cycle, chromosome segregation and thus cell ploidy [37]. In colorectal tumorigenesis, APC has never been proposed as a useful prognostic or predictive marker capable of differentiating between patients; thus, changes in APC status currently have no role in clinical practice. Loss of the p-arm of chromosome 8 is frequently observed in breast, prostate, and other types of cancers, and among the genes located close to D8S264 is MYOM2, whose expression is downregulated in breast cancer [38]. Finally, D20S107 is close to DNA topoisomerase I, a well-established molecular target of anticancer drugs, such as camptothecin derivatives, which are approved by the FDA for the treatment of colorectal tumors. Topoisomerase I levels are not predictive of drug cytotoxicity; rather, parameters downstream of the cleavable complexes are critical for the cytotoxicity of camptothecin [39].

### Identification of putative prognostic markers of survival

The survival study identified three potential survival prognostic markers for tumors without preoperative treatment at loci D1S197, D5S430, and D14S65. From a fundamental point of view, identification of the genes targeted by these markers would be of interest to understand how and which cellular alterations could lead a tumor to spread over metastasis sites. For instance, locus D1S197 is located on 1p32, a region that is deleted in many tumors, notably in neuroblastomas, colorectal and gastric cancers, and multiple endocrine neoplasia (MEN 2) [40]. A tumor suppressor gene called FAS-associated factor 1 (FAF1) localized in this region is involved in the regulation of apoptosis, NFκB activity [41], and neuronal cell survival [42], and its downregulation may contribute to multiple aspects of tumorigenesis. These data could justify that tumors with AIs, and thus carrying a likely deletion at this locus, may have a worse prognosis than tumors with a normal locus. Locus D14S65 targets region 14q32.2, in which vaccinia-related kinase 1 (VRK1) is localized. This gene is implicated in cell metabolism and cell proliferation, and its expression is increased in dividing cells. Some studies have reported that the VRK1 protein is necessary for exit from G0, and the loss of VRK1 could result in a block of cell cycle progression in G1/S [43]. Thus, patients which tumors have a normal D14S65 locus might have a worse prognosis than patients which tumors have this locus altered. Finally, D5S430 targets, among others, the phosphatidylinositol-specific phospholipase C, X domain containing 3 (PLCXD3) gene, for which no function in carcinogenesis has been reported thus far. Of course, only real-time PCR or CGH analyses would help to ascertain valid candidate genes.

### "Field effect" theory and colorectal carcinogenesis

The concept of cancer field effect has been proposed to explain the occurrence of genetic and epigenetic mosaicism in cancer precursor tissues [44]. According to this model, histologically normal adjacent tissue surrounding the tumor should have at least some, but not all, of the genetic alterations that are present in the fully developed cancer. These genetic alterations have been described in many precancerous cells of various organs (lung, breast, oesophagus, colorectal cancers...) and as proposed by Heaphy et al. [45], could include AI as shown in histologically normal breast tissues adjacent to the tumors. This implies that there might be a reservoir of genetically unstable cell clones within histologically normal tissue, representing a fertile ground for tumor development. The existence of these fields of genetically altered cells, appearing histologically normal and disease-free, is consistent with the hypothesis that genomic instability arises early in tumorigenesis. Thus, it could be relevant to study the status of the D1S197, D5S430 and D14S65 loci in normal adjacent tissue of rectal tumors. These loci might participate to the early step of rectal tumor carcinogenesis.

### Clustering of rectal cancer without preoperative treatment

The two-way hierarchical analysis clustered the rectal tumors without preoperative treatment into three clusters, CIN-VL, CIN-L, and CIN-H, according to the mean global AI frequency and loci associations. This repartition corroborated the clustering of colon tumors previously described by Weber et al. [23] as well as the alterations at microsatellites D18S61, D8S264, D8S283, and TP53, which are considered as early events in colorectal carcinogenesis [46]. In addition, there was no correlation between the tumor stage and any of the CIN-VL, CIN-L, and CIN-H clusters, and the survival analysis did not reveal any significant differences between clusters.

### Clustering of rectal and left colon tumors

To assess the similarity of genomic alterations in left colon and rectal tumors without preoperative treatment, allelotyping data from both locations were clustered together. The hierarchical cluster maintained the three clusters based on the mean AI frequency, and the tumors of the two origins did not segregate. However, despite a common panel of alterations on 8p, 18q, 17p, 20q and no significant differences in the AI frequency at each locus, we found that the loci that were associated as clusters in left colon tumors differed from those in rectal tumors, suggesting that rectal and left tumors should be considered as separate entities. Similarly, studies that investigated the mutational frequencies of K-Ras and APC or β-catenin and TP53 found differences between rectal and left colon tumors [11,47]. In contrast, Slattery et al. [12] showed that rectal and distal colon tumors share similar mutational frequencies for p53, K-Ras mutations and for the CpG island methylator phenotype, but differed from proximal colon tumors.

## Conclusions

We evidenced that tumor progression in rectal carcinomas, instead of being related to the frequency of genomic alterations at 33 chromosomal loci as assessed by allelotyping, could rather result from the disrupted functions of the locus-associated genes specifying a capacity to disseminate. Our data also identified some potential prognostic genomic markers, for some of which the associated genes remain to be identified. Their real implication as markers will require validation by a quantitative method, such as real-time PCR, for a larger cohort of rectal tumors. Finally, the pattern of alterations in rectal tumors differ from those in colon tumors reinforcing the concept that rectal and left tumors are separate entities.

## Competing interests

The authors declare that they have no competing interests.

## Authors' contributions

BR carried out data acquisition, analysis, data interpretation and manuscript preparation. AN, validated the histopathological parameters of the tumors. NM performed the clustering and some of the statistical analyses. CB and SR performed surgery and collected the tumor samples. AS participated in allelotyping data analysis. MPG and DG participated in the study design. DG critically revised the manuscript and gave the final approval of the version to be published. All authors read and approved the final manuscript.

## Pre-publication history

The pre-publication history for this paper can be accessed here:

http://www.biomedcentral.com/1471-2407/10/561/prepub
